# Photodegradation of naphthalene over Fe_3_O_4_ under visible light irradiation

**DOI:** 10.1098/rsos.181779

**Published:** 2019-01-30

**Authors:** Jiawei Zhang, Shanshan Fan, Bin Lu, Qinghai Cai, Jingxiang Zhao, Shuying Zang

**Affiliations:** Key Lab of Remote Sensing Monitoring of Geographic Environment, College of Heilongjiang Province, School of Chemistry and Chemical Engineering, Harbin Normal University, No. 1 Shida Road Limin development Zone, Harbin 150025, People's Republic of China

**Keywords:** naphthalene, photodegradation, nano Fe_3_O_4_ crystalline, visible light irradiation

## Abstract

Using FeCl_3_ and FeSO_4_ as precursors, Fe_3_O_4_ were prepared by co-precipitation method via FeCl_3_ and FeSO_4_ aqueous solutions successively added to NaOH solution. The sample was proved by X-ray powder diffraction, transmission electron microscope, ultraviolet–visible spectrophotometry and magnetic measurement. The results showed that the prepared Fe_3_O_4_ material was composed of an average diameter of about 15 nm particles and nano rods with well-crystallized magnetite and stronger superparamagnetic, getting a saturation magnetization of 49.5 emu g^−1^. This Fe_3_O_4_ material was found to be an effective catalyst for photodegradation of naphthalene with or without H_2_O_2_ under visible light irradiation, getting 81.1% and 74.3% degradation rate in these two cases, respectively. The degradation pathway in the absence and presence of H_2_O_2_ was analysed via measurement of the distribution of degradation products by GC-MS and adsorption of reactants on the surface of the catalyst by *in situ* DRIFTS spectra.

## 1. Introduction

Polycyclic aromatic hydrocarbons (PAHs) that consist of two or more condensed benzene rings are primarily formed from the incomplete combustion or pyrolysis of organic materials such as petroleum, gas, coal and wood [1]. These compounds have recently attracted a lot of attention in studies on water, soil and air pollution due to their highly carcinogenic, mutagenic and teratogenic potential [2]. The removal of such substances from the environment can be achieved using various methods, including physical, biological and chemical [3–5]. In the case of removing PAHs from aqueous systems, a physical process such as volatilization and adsorption can greatly reduce the amount of PAHs in the aqueous solution. However, this method does not completely solve the problems of PAHs pollution because of their unavailability for degradation of these pollutants. Besides, the conventionally biological method makes a limited contribution to the removal of PAHs from the aqueous solution due to the biorecalcitrant, toxic nature and low aqueous solubility of PAHs. Therefore, chemical processes have been used as alternatives to physical and biological ones because the former has greater potential for efficient removal of these pollutants from the aqueous system. Among the chemical methods, the removal of PAHs via photodegradation catalysed by heterogeneous semiconductor catalysts has been widely developed. These semiconductor catalysts involving TiO_2_, ZnO, SnO, WO_3_, Al_2_O_3_ and their modified nanomaterials exhibited efficiently photocatalytic activities for degradation of PAHs [6–9]. In particular, studies on modified or doped TiO_2_ nanomaterials have been widely conducted due to the pure TiO_2_ being significantly limited by its wide band gap, low light-application efficiency and high electron-holes recombination [10–12]. In recent years, catalytic and photocatalytic activities of iron oxides have been widely investigated because the iron oxides exhibit good performance, environmentally benign character and low cost. Yang *et al.* addressed the synthesis of α-Fe_2_O_3_ nanoparticles using the hydrothermal method and the synthesized α-Fe_2_O_3_ nanoparticles with smaller crystalline size show higher photocatalytic degradation efficiency than those of α-Fe_2_O_3_ powders with larger crystalline size [13]. Furthermore, a mixture of α-Fe_2_O_3_ and Fe_3_O_4_ exhibits better photocatalytic activity than that of pure α-Fe_2_O_3_ [14]. The higher activity of the mixed phase sample was likely attributed to the higher transfer of electrons and holes generated on the surface of α-Fe_2_O_3_ to the valence band of Fe_3_O_4_, which limits the recombination rate of electrons and holes [15]. Besides, Iron oxide nanocrystallites are active and selective catalysts for oxidation of aromatic compounds [16–18]. In the oxidation process using Fe_3_O_4_ as a catalyst, Fe^3+^ and Fe^2+^ participate in the catalytic oxidation cycle by interaction with the oxidant and it displays efficient catalytic activity [19–21]. However, few reports on the distribution of degradation products during the degradation of PAHs over iron oxides were found. So far, Fe_3_O_4_ nanoparticles are prepared by several methods, such as co-precipitation, precipitation in microemulsions, solvothermal reactions and hydrothermal method etc. [22–26]. Among them, the chemical precipitation is widely used due to its simple and practical nature. Usually, Fe_3_O_4_ nanoparticles are prepared by an aging stoichiometric mixture of ferrous [(FeCl_2_, FeSO_4_ or Fe(NO_3_)_2_] and ferric salts [(FeCl_3_, Fe_2_(SO_4_)_3_ or Fe(NO_3_)_3_] in aqueous medium and this process must have been carried out in a nitrogen atmosphere so as to prevent the Fe_3_O_4_ from being oxidized.

In view of these findings, nanosized Fe_3_O_4_ crystalline particles were prepared via an alternative method using FeCl_3_ and FeSO_4_ solutions successively dropwised in NaOH solution in order to maintain a basic medium for preventing FeO from being oxidized to Fe_2_O_3_. The photocatalytic performance of the prepared Fe_3_O_4_ nanoparticles for degradation of naphthalene in aqueous solution with or without H_2_O_2_ as oxidant were estimated and it displayed highly efficient photocatalytic activity. The results are disclosed in this work.

## 2. Experimental

### 2.1. Preparation of the catalysts

Iron oxide was prepared by the following procedure: a three-necked flask filled with 84 g of NaOH aqueous solution (1.25 mol l^−1^) with pH = 13.3 was put in a tank with ultrasonics and 65.4 g of FeCl_3_ aqueous solution (0.33 mol l^−1^) was dropwise added to the NaOH solution under ultrasonics and stirring. After adding, the reaction mixture was further ultrasonically treated for 40 min, the pH value of the mixture being changed from 13.3 to 12.8. The mixture was then heated in a water bath to 80°C, followed by dropwise addition of 62.8 g of FeSO_4_ aqueous solution (0.17 mol l^−1^) to this mixture with pH = 12.3, the temperature being changed to about 78°C. The mixture was continuously heated to 80°C and stirred at this temperature for 4 h until its pH value reached 12.1. After cooling down, the sol solution was filtered to remove the filtrate. The obtained gel was washed with anhydrous ethanol and deionized water three times, respectively, until the washing solution reached about pH = 7. Thereafter, it was dried at 50°C to attain Fe_3_O_4_.

### 2.2. Catalyst characterization

The X-ray powder diffraction (XRD) pattern of the sample was performed on a Bruker-D8 advance X-ray diffractometer with Cu K*_α_* radiation (40 kV and 36 mA). The morphology and particle size of the samples were observed by an FEI Tecnai F20 transmission electron microscope (TEM). The magnetic measurement was performed on a Vibration Sample Magnetometer (Lake Shore 7410). The diffuse reflectance ultraviolet–visible spectrum for the sample was recorded on a CARY 4000 UV–vis spectrometer.

*In situ* DRIFTS spectra were measured by an FT-IR spectrometer (Brucker TENSOR II) equipped with a diffuse reflectance optics accessory (Harrick Scientific products Inc.). To clean the catalyst surface, the iron oxide sample was thermally pretreated by heating to 50°C for 2 h *in vacuo* prior to acquisition of DRIFTS spectra. Thereafter, the build-up of naphthalene and/or H_2_O_2_ species onto the catalyst surface was performed by taking them along with high pure N_2_ gas (purity 99.99%) for 2 h, followed by evacuation from the system to about 9.8 × 10^−4^ Pa. After that, the DRIFTS spectra of surface species at 50°C were measured half an hour under *vacuo* conditions.

### 2.3. Catalytic test and analytic procedure

An amount of 10 ml of naphthalene solution (0.2 mmol of naphthalene dissolved in 100 ml ethanol + 100 ml deionized water), 90 ml of deionized water and 0.3 g of Fe_3_O_4_ were added to a quartz columnar reactor (placed in a water-bath to maintain the temperature at 23–25°C) fitted with a magnetic stirrer. The reaction mixture with pH = 6.96 was irradiated by *λ* = 400–780 nm visible light (HSX-F/UV300, using a VISREF780 filter, Beijing NBet science & technology Ltd Co.) under stirring for 8 h (intensity of illumination 2.7 × 10^5^ LX). The light source was close to the wall of the reactor. At the end of the reaction (the mixture solution with pH = 7.22; when using H_2_O_2_ oxidant the pH changed from 3.50 initially to 4.50 after the reaction.), the catalyst was separated from the reaction mixture by a magnet. The sample solution was analysed by a Lamda-35 ultraviolet spectrometer (PerkinElmer Company) to determine degradation rate. The sample solution was extracted by 10 ml of CH_2_Cl_2_ three times and the upper phase was analysed by GC-MS (Agilent GC7890A-MS5975C) using an HP-5 column (30 m × 0.32 mm × 0.25 um) to identify the degradation products. The degradation rate was calculated by the following formula:

$$\mathrm{rate}=\frac{C_{0}-C_{t}}{C_{0}}\times 100\text{\%},$$

where *C*_0_ is the initial concentration of naphthalene, *C_t_* is the concentration of naphthalene at a certain time.

## 3. Results and discussion

### 3.1. Preparation and characterization

In our previous work [27], Fe_3_O_4_ was prepared in an alternative way, using acid pickling waste as a precursor added to the basic solution to ensure the pH ≧ 8 during the preparation process, whereas in this case, Fe^3+^ (FeCl_3_) and Fe^2+^ (FeSO_4_) solutions were used as precursors in the absence of PEG-400 dispersant and Fe_3_O_4_ nanoparticles were successfully prepared at pH = 12.1 for precipitation by this simple method.

**Figure 1.**
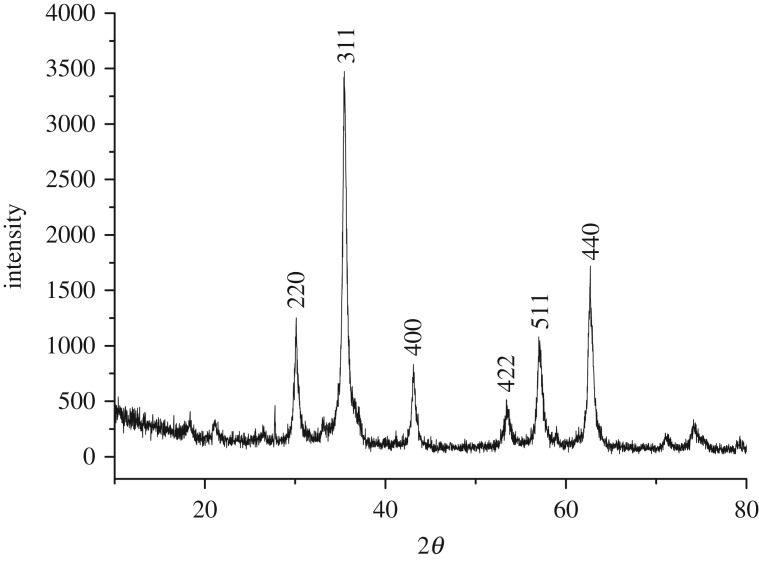
XRD pattern of Fe_3_O_4_.

**Figure 2.**
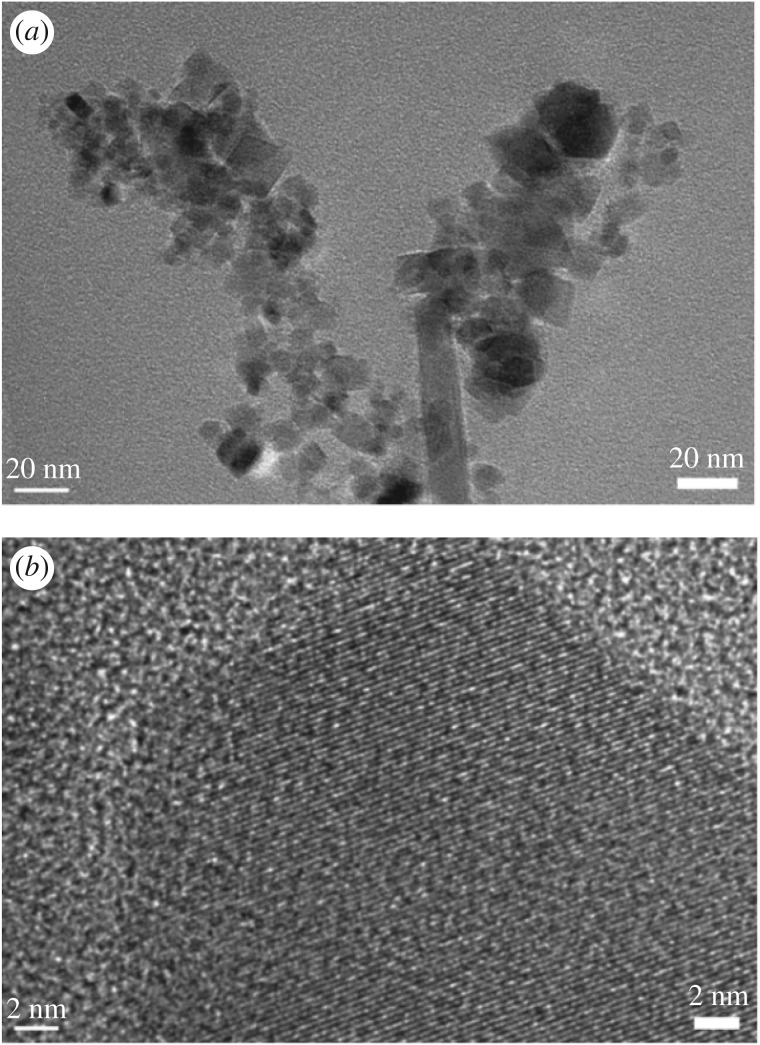
TEM images of Fe_3_O_4_ materials.

**Figure 3.**
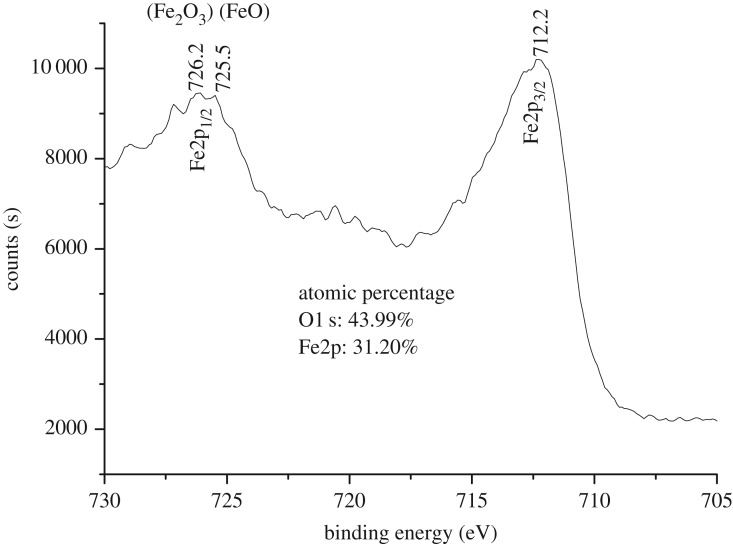
XPS spectrum of Fe_3_O_4_.

**Figure 4.**
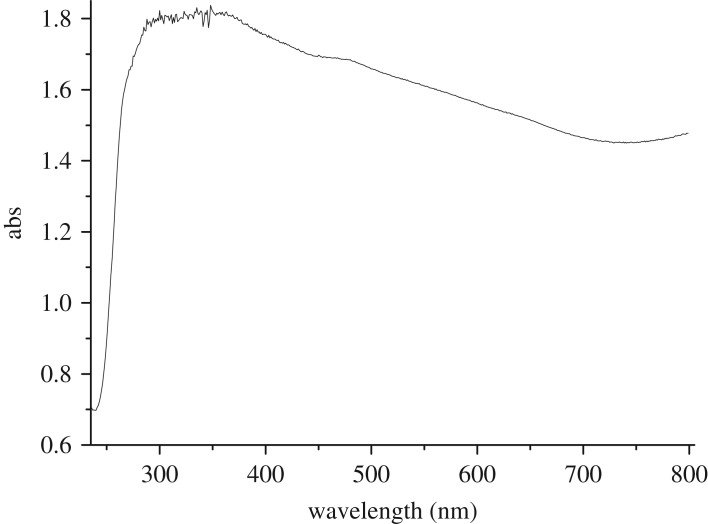
Diffuse reflectance UV–vis spectrum of Fe_3_O_4_.

X-ray powder diffraction of iron oxide sample was recorded in 2*θ* range of 10–80°. It could be seen from the XRD pattern of the sample shown in figure 1 that typical diffraction peaks at 2*θ* of 30.1, 35.4, 43.1, 53.4, 57.0 and 62.7° corresponded to (220), (311), (400), (422), (511) and (440) lattice planes, respectively. No peak from other impurities were found in this image, suggesting that close to single phase Fe_3_O_4_ was successfully prepared and all the diffraction peaks were perfectly indexed, which was in agreement with the data of the cubic phase Fe_3_O_4_ (magnetite JCPDS 19–0629) [28]. The average crystalline size of the sample was calculated according to the Scherrer equation and the estimated particle size approached 14.4 nm. This estimation was basically in agreement with one observed in the TEM image (figure 2*a*). As seen from the image, particles of about 15 nm in size and nano rods of about 15 nm in diameter were observed. However, the nanostructured material was further examined with high-resolution TEM. A typical HRTEM image of nanostructured Fe_3_O_4_ was obtained as shown in figure 2*b*. The lattice fringes in the image corresponding to a set of atomic planes within the particles were clearly observed. X-ray photoelectron spectroscopy of Fe_3_O_4_ was shown in figure 3. As seen in the figure, a typical peak corresponding to Fe_3_O_4_ was observed at 712.2 eV, which was basically in accordance with 711.4 eV for pure Fe_3_O_4_ in reported literature [29]. Obviously, a set of distinct binding energies were observed for the Fe 2p_1/2_ spectra, one at 725.5 eV and the other at 726.2 eV, which were assigned to characteristics of FeO and Fe_2_O_3_, respectively. The atomic ratio of O to Fe was estimated to be 4 : 3 by XPS. This also elucidated that pure Fe_3_O_4_ was successfully obtained. Besides, the higher visible light absorption of the Fe_3_O_4_ sample was found in the range of 400–780 nm, as shown in UV–vis spectrum (figure 4). This indicated that Fe_3_O_4_ could be activated by visible light.

**Figure 5.**
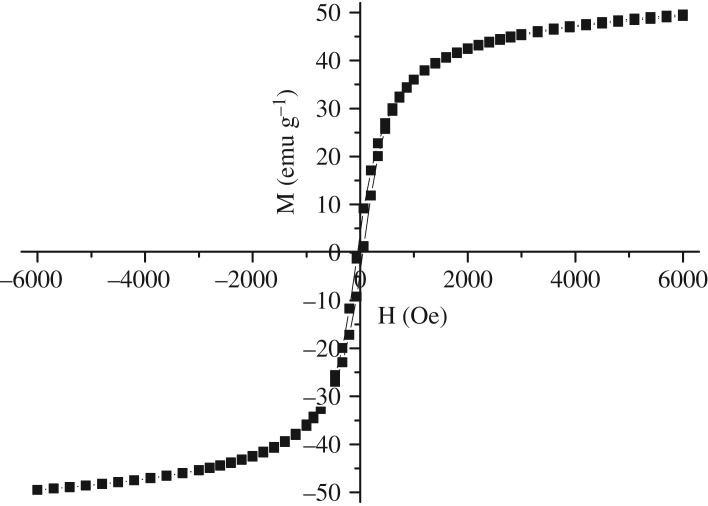
Magnetic hysteresis curve for Fe_3_O_4_ sample.

The curve of the magnetization versus magnetic field for the prepared sample was shown in figure 5. Because an M-H loop was found in the curve during the external field cycle scanning from −6 kOe to 6 kOe, the hysteresis was observed. The figure shows that the Fe_3_O_4_ crystalline was superparamagnetic [30] with the highest magnetization saturation M(s) of 49.5 emu g^−1^, the coercivity value H(c) of 50.1 Oe and the remanence value M(r) of 3.9 emu g^−1^. This suggested that the Fe_3_O_4_ particles could act as a magnetically separable catalyst.

### 3.2. Degradation of naphthalene

Table 1 presents the photocatalytic activity of Fe_3_O_4_ using various preparation methods. It was clear that Fe_3_O_4_ achieved highly photocatalytic activity, exhibiting 74.3% degradation rate in the absence of H_2_O_2_. Fe_3_O_4(N)_ prepared by NH_3_·H_2_O substituting for NaOH solution displayed lower activity, as did the Fe_3_O_4(7)_, Fe_3_O_4(c)_ and α-Fe_2_O_3_. As a consequence, the Fe_3_O_4_ prepared by FeCl_3_ and FeSO_4_ aqueous solutions successively added to NaOH solution and reaction at 80°C possessed higher photocatalytic activity, which was applied as a catalyst in the next sections.

**Table 1.** Photocatalytic activity of various iron oxides. N-NH_3_·H_2_O substituting for NaOH solution during preparation; 7–70°C; c-co-precipitation.

Fe_y_O_x_	Fe_3_O_4_	Fe_3_O_4(N)_	Fe_3_O_4(7)_	Fe_3_O_4 (c)_	α-Fe_2_O_3_
rate (%)	74.3	40.4	34.2	57.3	28.2

**Figure 6.**
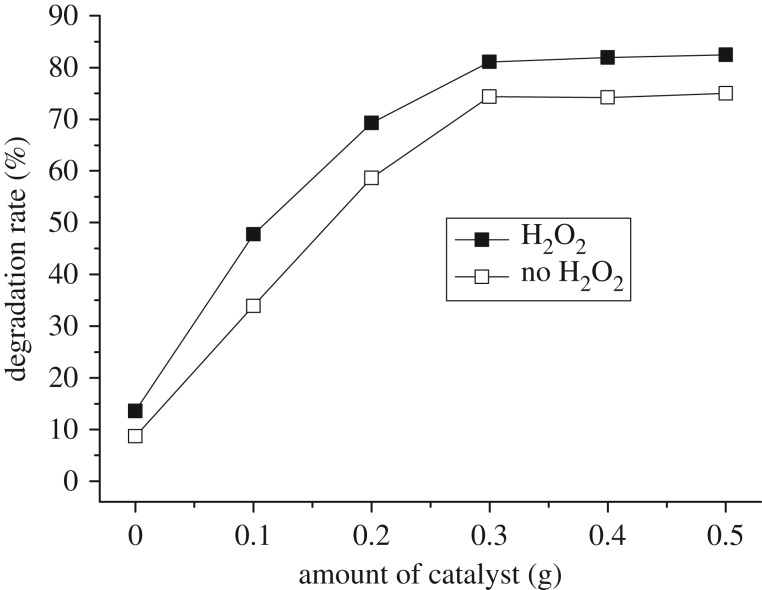
Effect of catalyst dose on degradation without and with H_2_O_2._ Reaction conditions: initial concentration 0.1 mmol l^−1^, time 8 h, H_2_O_2_ 8 ml.

Catalytic activity of Fe_3_O_4_ without visible light irradiation was analysed in the presence of H_2_O_2_. Degradation of naphthalene was performed with 10.0% degradation rate, which could be enhanced under visible light irradiation. The reference experiments in the absence of Fe_3_O_4_ under visible light irradiation revealed low rates of 13.6% with H_2_O_2_ and 8.3% without H_2_O_2_, respectively (figure 6). When using Fe_3_O_4_ as a catalyst the photodegradation was greatly promoted. It was therefore evident that Fe_3_O_4_ could effectively photocatalyse degradation of naphthalene and the addition of H_2_O_2_ into this system indeed enhanced the photocatalytic degradation.

However, the catalytic activity was increased as the amount of the Fe_3_O_4_ increased with and/or without H_2_O_2_. As the amount of the catalyst approached 0.3 g, the rates reached 74.3% in the absence of H_2_O_2_ and 81.1% in the presence of H_2_O_2_, respectively. Thereafter, the rates held constant in these two cases as the amount was increased from 0.3 g to 0.5 g.

**Figure 7.**
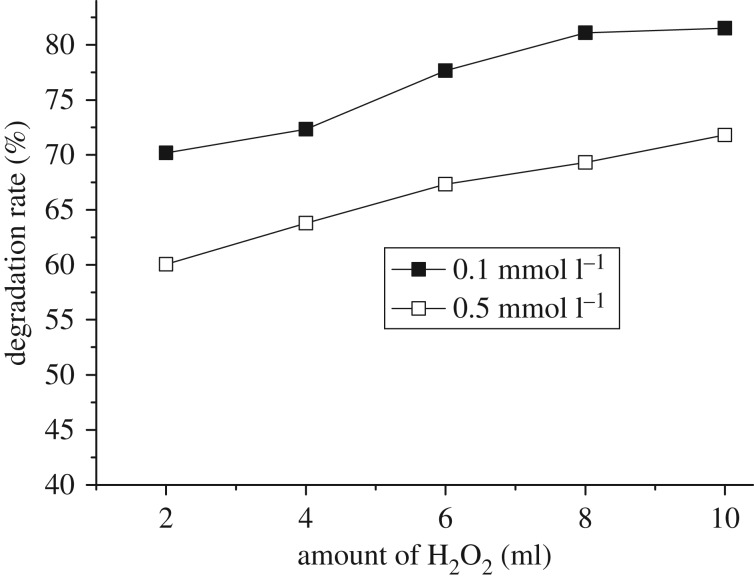
Effect of H_2_O_2_ amount on the degradation. Reaction conditions: initial concentration 0.1 and 0.5 mmol l^−1^, time 8 h, amount of catalyst 0.3 g.

Besides, increasing the amount of added H_2_O_2_ could evidently promote the degradation rate of naphthalene at 0.1 and 0.5 mmol l^−1^ of initial concentrations. As the added amount of H_2_O_2_ reached 8 ml, the rates approached 69.5% for 0.5 mmol l^−1^ and 81.1% for 0.1 mmol l^−1^ of initial concentration, respectively; and then continuously increasing the amount of H_2_O_2_ led to slight promotion of the degradation rate, as shown in figure 7.

**Figure 8.**
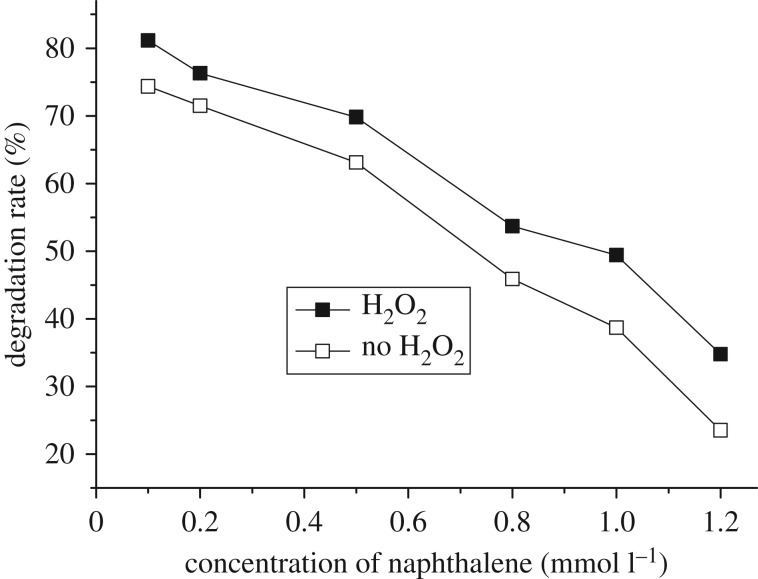
Effect of naphthalene concentration on degradation without and with H_2_O_2_. Reaction conditions: time 8 h, amount of catalyst 0.3 g, H_2_O_2_ 8 ml.

The degradation of naphthalene at initial concentration of 0.1–0.5 mmol l^−1^ was investigated and the results were depicted in figure 8. The rate was increased as the concentration of naphthalene in ethanol–water solution decreased, resulting in 74.3% of degradation rate at a concentration of 0.1 mmol l^−1^ in the absence of H_2_O_2_. Moreover, the addition of H_2_O_2_ into this system also enhanced the photocatalytic degradation, the rate was promoted to 81.1% at the same concentration of naphthalene. The rate of increase for the two cases, photocatalysis and photocatalytic oxidation, were coincident with each other, indicating that the enhancing power of H_2_O_2_ was based on the photocatalysis across the entire range of concentrations.

**Figure 9.**
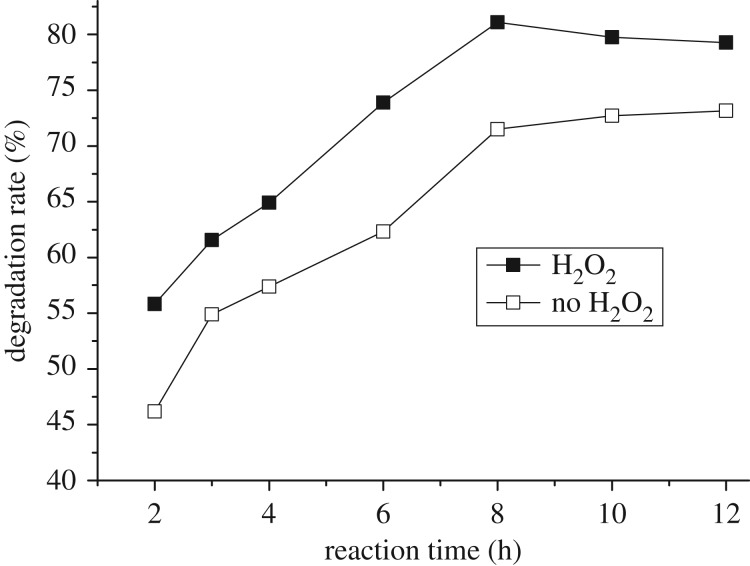
Effect of reaction time on the degradation without and with H_2_O_2_. Reaction conditions: initial concentration 0.1 mmol l^−1^, amount of catalyst 0.3 g, H_2_O_2_ 8 ml.

**Figure 10.**
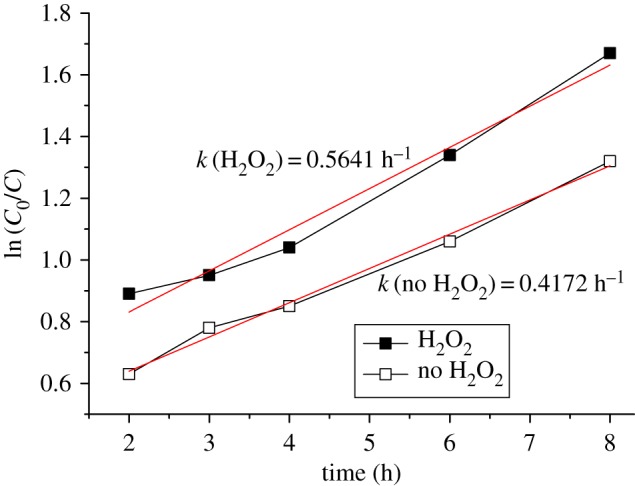
Kinetic curves for photodegradation of naphthalene.

The catalytic degradation-time profile of the degradation reaction of naphthalene using Fe_3_O_4_ as a catalyst under visible irradiation in the presence and/or absence of H_2_O_2_ was shown in figure 9. The degradation rate was increased in the time range from 2 to 8 h, exhibiting a 74.3% rate in the absence of H_2_O_2_ and a 81.5% rate in the presence of H_2_O_2_ at 8 h. As the reaction proceeded beyond 8 h, the degradation rates of naphthalene basically remained unchangeable with the reaction proceeding from 8 to 12 h in these two cases, implying that the reaction achieved thermodynamic equilibrium at that time. Besides, the photocatalytic degradation of naphthalene was synchronized with the reaction time in the absence or presence of H_2_O_2_. As compared with reported works on the photodegradation of naphthalene using TiO_2_ supported materials and Ce, N and P tri-doped TiO_2_/AC as catalysts [12,31], the Fe_3_O_4_ displayed higher activity than the TiO_2_ supported catalyst and almost the same photodegradation efficiency as the doped TiO_2_/AC. In addition, according to the experimental data, as shown in figure 9, a plot of ln (*C*/*C*_0_) versus time was obtained (figure 10), where *C* and *C*_0_ are the concentration of naphthalene at certain time *t* and the initial one, respectively. These curves for ln (*C*/*C*_0_) versus time were linear, suggesting that the photodegradation reaction is clearly quasi-first order in naphthalene whether in the presence or absence of H_2_O_2_. The first-order rate constants calculated from the plots are *k* (H_2_O_2_) = 0.5641 h^−1^ for photocatalytic oxidation degradation and *k* (no H_2_O_2_) = 0.4172 h^−1^ for photodegradation in the absence of H_2_O_2_, respectively. These findings also meant that photogradation of naphthalene with H_2_O_2_ is faster than the system without H_2_O_2._

**Figure 11.**
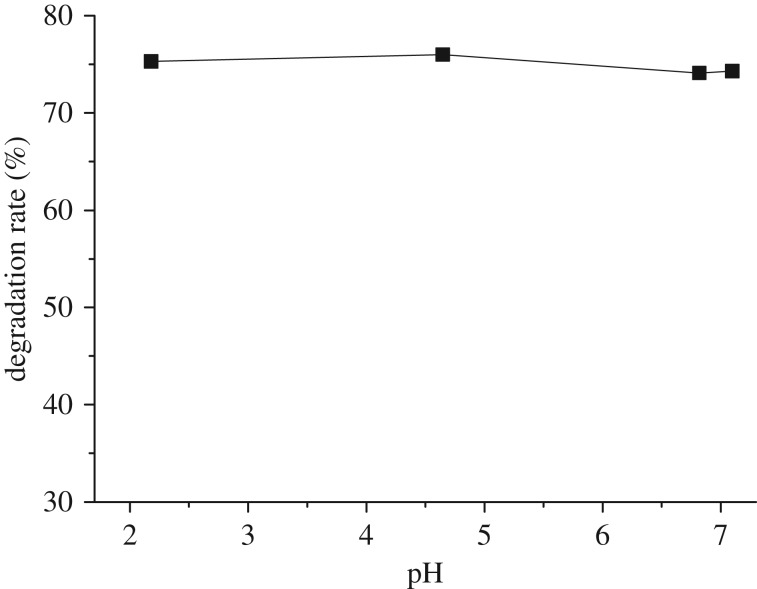
Effect of pH value on the degradation without H_2_O_2_. Reaction conditions: initial concentration 0.1 mmol l^−1^, amount of catalyst 0.3 g, H_2_O_2_ 8 ml.

Figure 11 displays the effect of pH values of the solution on the degradation without H_2_O_2_. It was clear that the pH values did not affect the degradation of naphthalene.

### 3.3. *In situ* DRIFTS spectra and degradation mechanism

**Figure 12.**
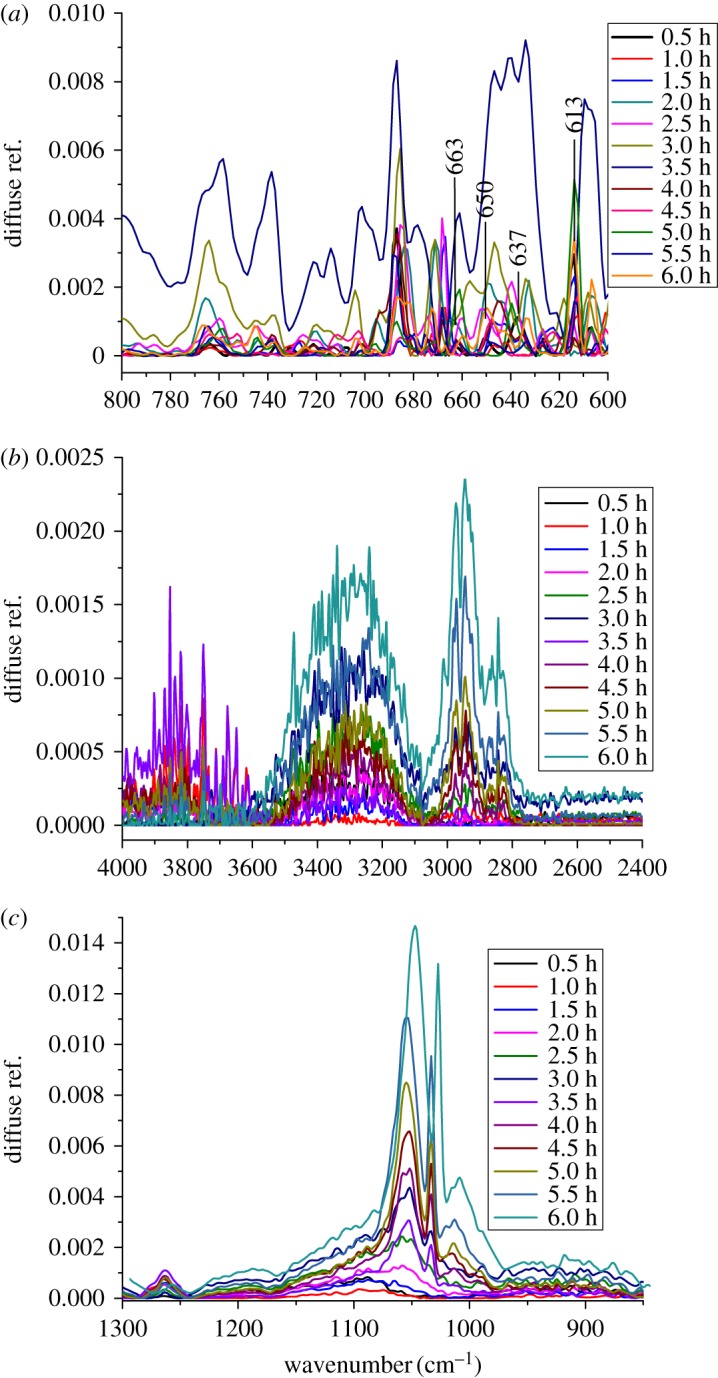
*In situ* DRIFTS spectra of naphthalene and H_2_O_2_ on Fe_3_O_4_ surface.

**Figure 13.**
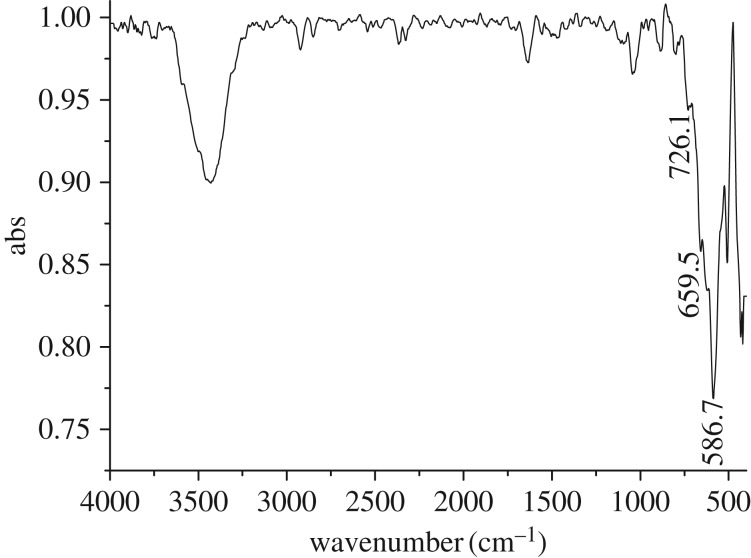
FT-IR spectrum of Fe_3_O_4_.

In order to explore the degradation mechanism, *in situ* DRIFTS spectra of the naphthalene and H_2_O_2_ adsorbed on the catalyst surface were measured at 50°C, as shown in figure 12. The characteristic band of Fe-O bond at 586.7 cm^−1^, shoulder bands at 659.5 and 726.1 cm^−1^ in the FT-IR spectrum of Fe_3_O_4_ catalyst (figure 13) were greatly weakened and it was likely divided into four bands at 613, 637, 650 and 663 cm^−1^ due to adsorption of the naphthalene and H_2_O_2_ on the catalyst surface by means of interacting with Fe^2+^ or Fe^3+^ species. The intensity of these bands was markedly increased to the strongest as the adsorption time reached 3.5 h, and it was then decreased from 3.5 to 6.0 h, as shown in figure 12*a*. This suggested the strong interaction between the reactants and Fe^2+^ or Fe^3+^ active sites on the surface of the catalyst. Synchronously, the characteristic bands around 2800–3000 cm^−1^ corresponding to C–H stretching vibration in CH_2_– or CH_3_– groups were enhanced as the time was extended until maximum intensity was achieved at a time 6 h (figure 12*b*), which indicated that the aromatic ring open in the naphthalene molecule likely took place on the catalyst surface to generate CH_3_– or CH_2_– groups. Besides, abundant free hydroxyl groups at 3500–3800 cm^−1^ and associated hydroxyls at 3100–3500 cm^−1^ such as ·OH and ·OOH radicals produced by interaction between H_2_O_2_ and Fe^2+^ or Fe^3+^ species were found on the catalyst surface. This process for the production of ·OH and ·OOH radicals proceeded through the following equations [30,32]:

3.1 $$Fe^{2+}+H_{2}O_{2}\to Fe^{3+}+OH^{-}+\cdot \mathrm{OH}$$

3.2 $$Fe^{3+}+H_{2}O_{2}\to Fe^{3+}+H^{+}+\cdot \mathrm{OOH}$$

3.3 $$Fe^{3+}+\cdot \mathrm{OOH}\to Fe^{2+}+H^{+}+O_{2}.$$

As the adsorption proceeded the intensity of these bands corresponding to free hydroxyls was first increased from 0.5 to 3.5 h and then significantly decreased beyond 3.5 h, implying the ·OH and ·OOH radicals were generated at the initial stage and consumed to oxidize naphthalene or other intermediates beyond 3.5 h. On the contrary, the intensity of the bands ascribed to associated hydroxyls was increased to the strongest at 6 h, which was likely related to conversion of free hydroxyls into associated ones on the surface. Likewise, the bands at 1000–1200 cm^−1^ attributed to stretching vibration of C–O–O bonds [33] were also increased remarkably (figure 12*c*). These findings suggested that the decrease in the intensity of free hydroxyls was likely ascribed to conversion of naphthalene interacting with ·OOH into hydroperoxy- and/or hydroxy-intermediates, the intermediates further converted into the degradated products.

**Scheme 1.**
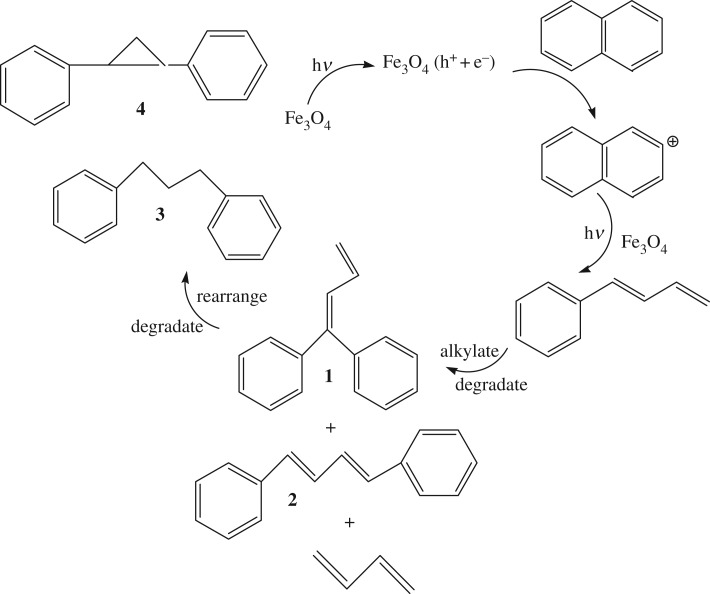
The proposed mechanism for degradation without H_2_O_2_.

On the other hand, the distribution of degradated products in the presence or absence of H_2_O_2_ were determined by GC-MS. Different degradation products were confirmed due to their different photocatalytic reactions in these two cases. As shown in Scheme 1, the photocatalytic degradation of naphthalene over Fe_3_O_4_ was carried out in the absence of H_2_O_2_, generating 1,2-diphenyl butadiene (**1**) or 1,4-diphenyl butadiene (**2**), 1,3-diphenyl propane (**3**) and 1,2-diphenyl cyclopropane (**4**) as the main products (their MS images and data are shown in the electronic supplementary material). This suggested that the degradation process was mainly carried out via aromatic ring-opening reaction in naphthalene molecules and continuous catalytic alkylation, degradation and rearrangement between the ring-opened products to form diphenyl compounds. According to the product distribution and the works reported in the literature, the mechanism for the degradation process was described as shown in scheme 1.

When Fe_3_O_4_ was irradiated, a hole and electron were, respectively, generated on its surface. Fe(III) transforms into Fe(II) after receiving an electron, and the oxidation process may proceed by one electron transfer leading to the formation of the naphthalene cation radical [34]. The strong adsorption of naphthalene on the Fe_3_O_4_ surface facilitates the electron transfer from the excited Fe(III)-Fe_3_O_4_ to organic molecules and such a transfer can initiate the photodegradation of naphthalene. Firstly, adsorbed naphthalene cation opened the aromatic ring to form phenyl butadiene under visible irradiation, then the phenyl butadiene undergoes alkylation with another phenyl butadiene molecule, followed by degradation catalysed by Lewis acid on the catalyst surface to generate **1**, **2** and butadiene; furthermore, the products **1** and **2** continuously perform degradation and/or rearrangement to form **3** and **4** catalysed by Lewis acidity on the catalyst surface.

**Scheme 2.**
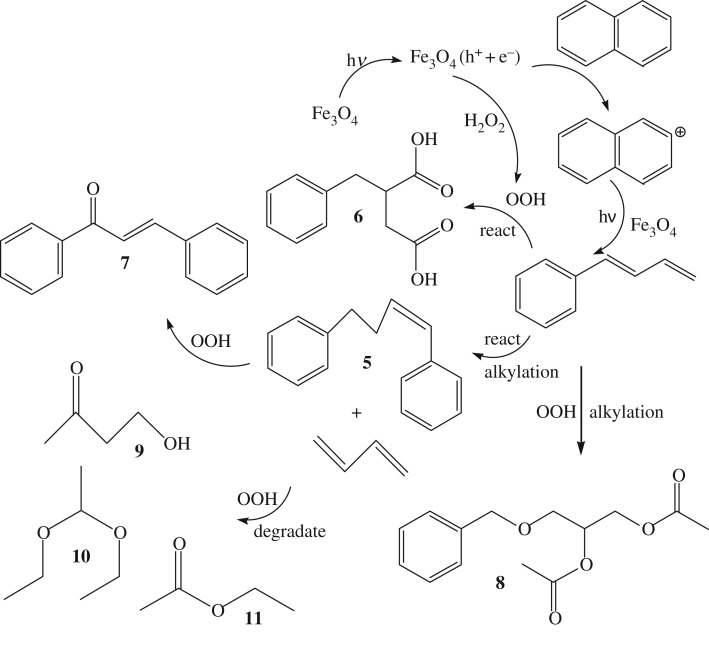
The proposed mechanism of degradation with H_2_O_2_.

In the presence of H_2_O_2_, the transient Fe(II) or Fe(II) in Fe_3_O_4_ was able to bind and reduce H_2_O_2_ to produce ·OOH and/or ·OH radicals with high redox potential, which has been experimentally proven by the reported works [35,36], as well as the above *in situ* DRIFTS spectra. This strongly suggested that the degradation process was carried out via photocatalysis and oxidative action of ·OOH and/or ·OH radicals. For this reason, the photocatalytic degradation and oxidation progress of naphthalene took place synchronously in the presence of H_2_O_2_. As a result, the degradation products were very different from the former due to the oxidizing action. The main products determined by GC-MS included 1,4-diphenylbutene-1 (**5**), benzyl succinic acid (**6**), 1,3-diphenyl acrylketone (**7**), (benzyl methyl ether)-ethyl diacetate (**8**) and hydroxyethyl methyl ketone (**9**), a small quantity of 2-ethoxy ethyl ether (**10**) and ethyl acetate (**11**) were also found in the reaction mixture as depicted in scheme 2 (Their MS images and data were as shown in the electronic supplementary material). The production of these products in the presence of H_2_O_2_ revealed that the photocatalytic degradation in this case also initiated from aromatic ring open, and then carried out the oxidation, alkylation and degradation of these ring-open products. Based on the product distribution and the *in situ* DRIFTS spectra analysis, the reaction mechanism was proposed as well, as shown in scheme 2.

Firstly, H_2_O_2_ and naphthalene were adsorbed on the surface of the catalyst via the interaction of the reactants with the active sites of Fe^2+^ or Fe^3+^ species. The adsorbed H_2_O_2_ molecule was oxidized by Fe^2+^ to form ·OOH and/or ·OH radicals on the surface. The surface conversion of adsorbed naphthalene took place to generate adsorbed ring-open product phenyl butadiene; then the ring-opened product carried out alkylation and degradation to form diphenyl butene **5** and butene through interaction with Lewis acids, followed by oxidation and alkylation of phenyl butadiene to generate the compound **6**, as well as the compound **8**, and oxidation of **5** to produce the product **7**. At the same time, the butadiene was degradated and oxidized to the compounds **9**, **10** and **11** acting through light irradiation and ·OOH or ·OH radicals. In addition, a small quantity of the compounds **10** and **11** possibly came from the oxidation and/or reaction between the degradated C1-C2 species or the oxidation and esterification of solvent ethanol.

### 3.4. The reusability of the catalyst

**Figure 14.**
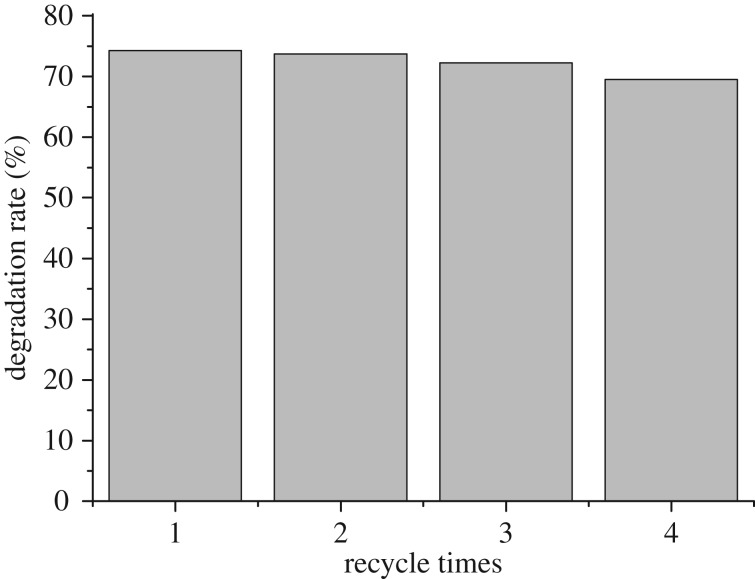
Reusability of Fe_3_O_4_.

**Figure 15.**
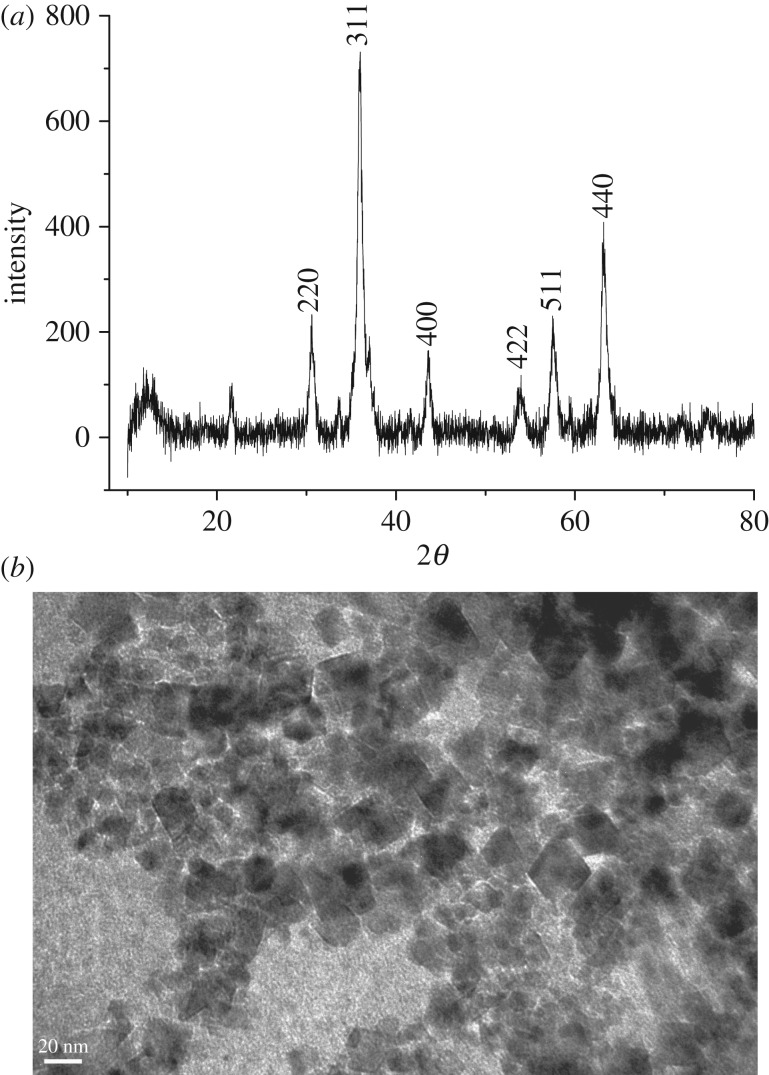
XRD pattern (up) and morphology (down) of the used catalyst (four times).

In order to further evaluate the photocatalytic performance of Fe_3_O_4_, recovery and recyling of the Fe_3_O_4_ were explored. The Fe_3_O_4_ was recovered by magnetic separation after each run, and then it was reused in the next run under the same reaction conditions. It was clear that the activity of the catalyst was almost unaffected even at the fifth run (figure 14). Moreover, the XRD pattern of the reused catalyst was the same as that of the fresh one four times, as shown in figure 15 (up). Also, the particle size of the reused Fe_3_O_4_ was almost unchangeable when compared with the fresh one, although slight aggregation was observed in figure 15 (down). These findings showed that the prepared Fe_3_O_4_ was highly stable at least within four repeated runs. The slightly lower activity for the fourth one was likely ascribed to loss of the catalyst amount during the process of recovery.

## 4. Conclusion

Nanosized Fe_3_O_4_ material was prepared by using FeCl_3_ and FeSO_4_ precursor aqueous solution successively dropwised in NaOH solution to maintain the stability of Fe_3_O_4_ in basic medium. This nanomaterial exhibited high photocatalytic activity for degradation of naphthalene in the absence or presence of H_2_O_2_, getting 74.3% and 81.5% of degradation rates, respectively, for the two cases. In the process, the photodegradation products involving diphenyl butadiene, diphenyl propane, benzyl succinic acid, 1,3-diphenyl acrylketone, (benzyl methyl ether)-ethyl diacetate and hydroxyethyl methyl ketone etc. were determined by GC-MS. *In situ* DRIFTS spectra of naphthalene and H_2_O_2_ adsorbing on the catalyst surface showed that high activity of Fe_3_O_4_ for photodegradation of naphthalene was ascribed to the production of hole-electron pairs and ·OOH radicals on the surface, which preceeded degradating or oxidizing to the adsorbed naphthalene. Based on *in situ* DRIFTS analysis and distribution of the degradation products, the degradation mechanism involving adsorption of naphthalene and H_2_O_2_, ring open in naphthalene molecules on the catalyst surface and/or oxidation of ring open intermediates was achieved. Besides, the Fe_3_O_4_ could be easily recovered and effectively reused.

## Supplementary Material

Supporting information

## Supplementary Material

Supporting information

## Data accessibility

The datasets supporting this article have been uploaded as part of the electronic supplementary material.
