# Supplementary material for: Photodegradation of naphthalene over Fe3O4 under visible light irradiation
Source: R Soc Open Sci. 2019 Jan 30;6(1):181779. doi: 10.1098/rsos.181779 (PMC6366235; doi:10.1098/rsos.181779)
Supplement: Supporting information [file rsos181779supp1.doc]

**Supporting information**

**Photodegradation of naphthalene over Fe_3_O_4_ under visible light**

**irradiation**

| Code | Intermediates | m/z^+^ |
| --- | --- | --- |
| **1** | 1,2-diphenyl butadiene | 91, 104, 117, 129, 193, 207 |
| **2** | 1,4-diphenyl butadiene | 32, 44, 65, 77, 91, 103, 115, 130, 165, 179, 194, 206 |
| **3** | 1,3-diphenyl propane | 40, 51, 77, 91, 101, 115, 128, 165, 178, 191, 206) |
| **4** | 1,2-diphenyl cyclopropane | 51, 65, 77, 91, 101, 115, 142, 165, 179, 191, 194 |
| **5** | 1,4-diphenylbutene-1 | 65, 77, 91, 104, 117, 129, 131 |
| **6** | benzyl succinic acid | 65, 77, 91, 103, 117, 129, 179, 194, 207 |
| **7** | 1,3-diphenyl acrylketone | 32, 45, 73, 91, 103, 115, 178, 193, 207, 219 |
| **8** | (benzyl methyl ether)-ethyl diacetate | 65, 77, 91, 104, 117, 194, 207, 267 |
| **9** | hydroxyethyl methyl ketone | 15, 31, 43, 61, 70, 88 |
| **10** | 2-ethoxy ethyl ether | 40, 51, 65, 77, 92, 105, 117 |
| **11** | ethyl acetate | 29, 43, 61, 70, 88 |

Jiawei Zhang, Shanshan Fan, Bin Lu, Qinghai Cai*, Jingxiang Zhao, Shuying Zang*

**Table SI1** Data of GC-MS measurement for reaction byproducts


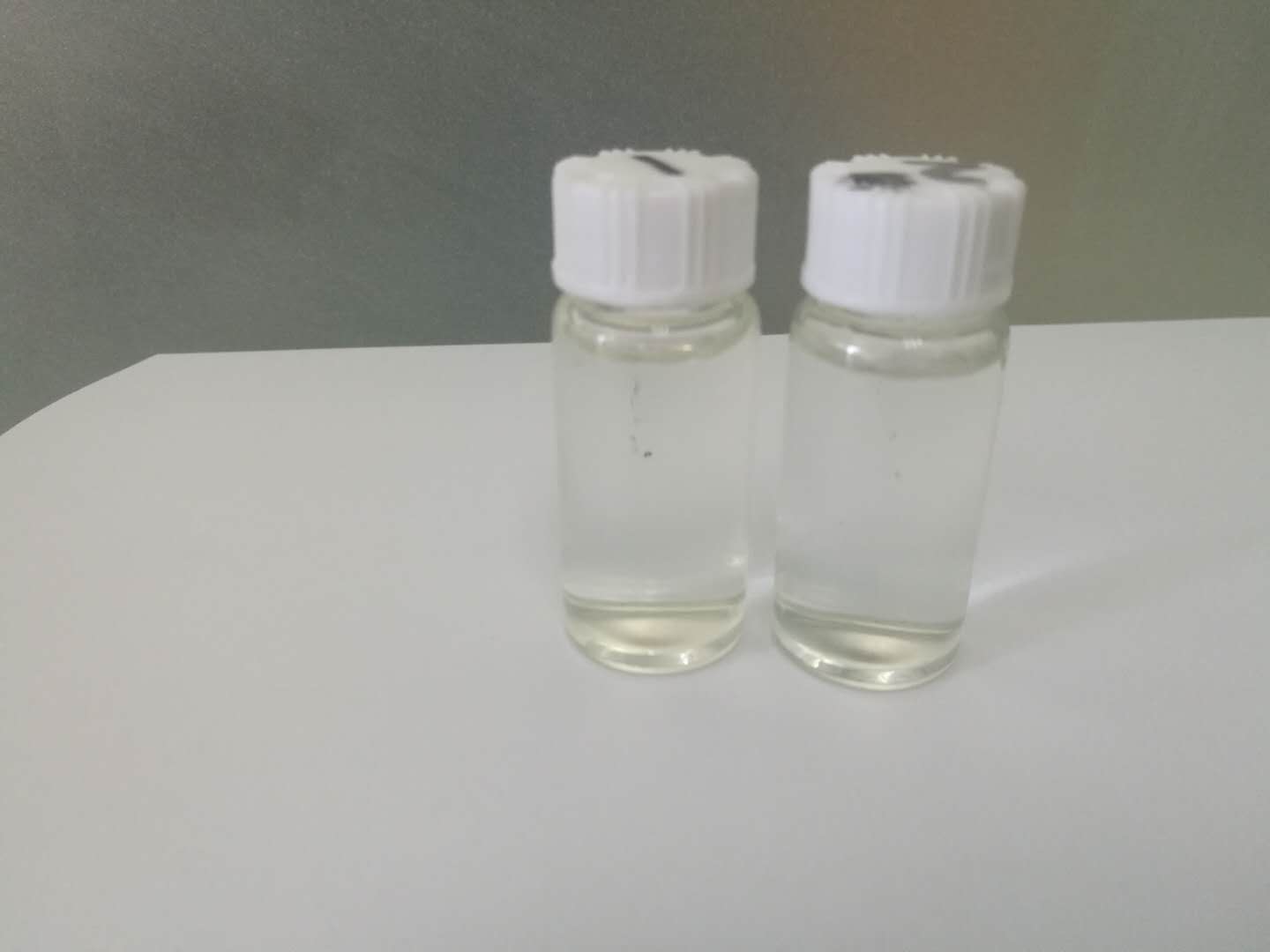


**Fig SI1** The sample solution before (2) and after (1) reaction
